# Supplementary material for: Mapping the microscale origins of magnetic resonance image contrast with subcellular diamond magnetometry
Source: Nat Commun. 2018 Jan 9;9:131. doi: 10.1038/s41467-017-02471-7 (PMC5760582; doi:10.1038/s41467-017-02471-7)
Supplement: Supplementary file 1 — Supplementary Information [file 41467_2017_2471_MOESM1_ESM.pdf]

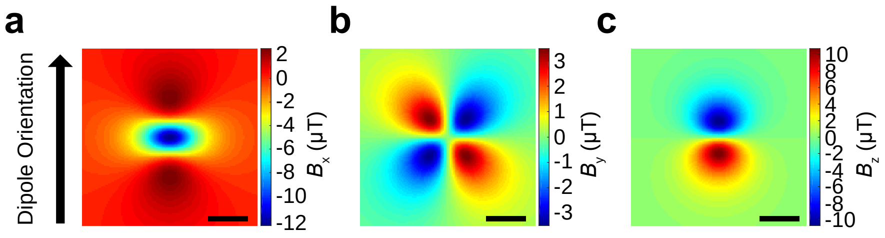

Supplementary Figure 1 - Simulated dipole fields. Simulated B_x (**a**), B_y (**b**), and B_z (**c**) field projections for a point dipole oriented towards the top of the image with a magnetic moment of $10^{-15} A \cdot m^2$. The x and y coordinates of the dipole are fixed at the center of the image and the dipole is spaced two μm above the plane of projection. As in the main text, x is defined along the dipole axis, z is defined out of the page, and y is defined to complete the normal basis. Scale bars are 2.5 μm.

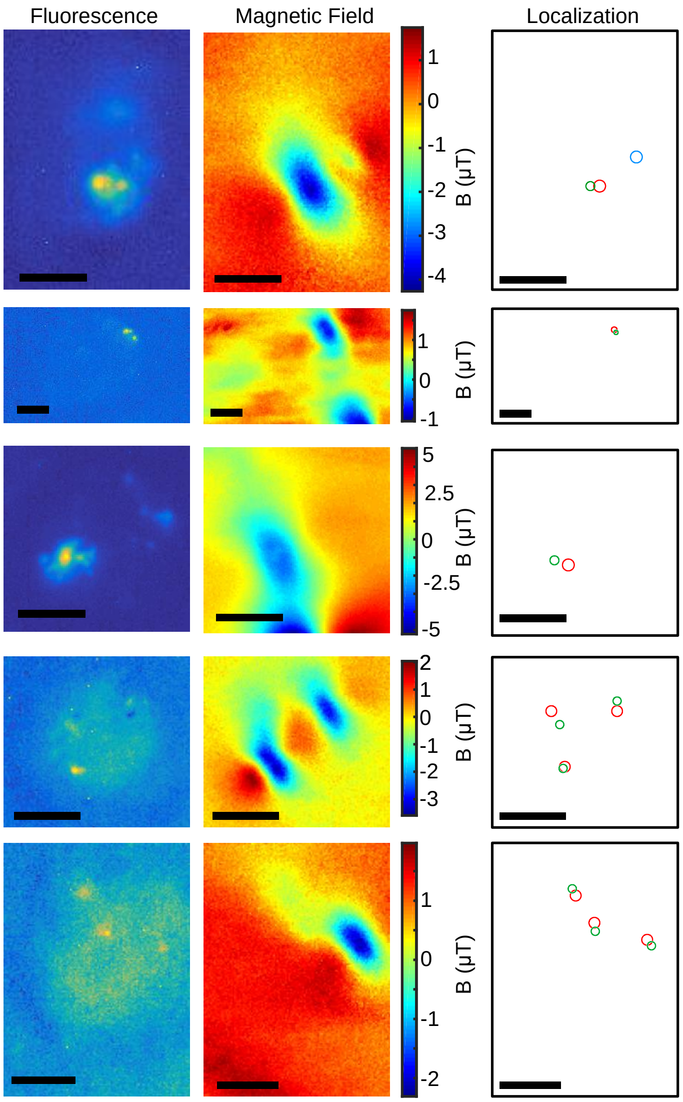

Supplementary Figure 2 - Magnetic–fluorescent colocalization. Fluorescence and magnetic images of fixed cells after the uptake of 200 nm (iron oxine nanoparticles) IONs labeled with Alexa 488 fluorescent dye. Magnetic localization (red circles) shows strong fidelity to the centroids of fluorescent images (green circles), with a mean offset of < 800 nm. Circle diameters are fixed to the diffraction limit for the magnetometry and Alexa 488 dye fluorescence respectively. The sole mismatch occurred in the top cell, where a second dipole was visible in the nitrogen vacancy (NV)-based image and localization, but there was no corresponding centroid in the Alexa 488 fluorescent image. Magnetic localization of this dipole is marked with blue. Fitting of this dipole revealed that it possessed the magnetic moment of a single nanoparticle, perhaps explaining the weak fluorescent signal. Scale bars are 5 µm.

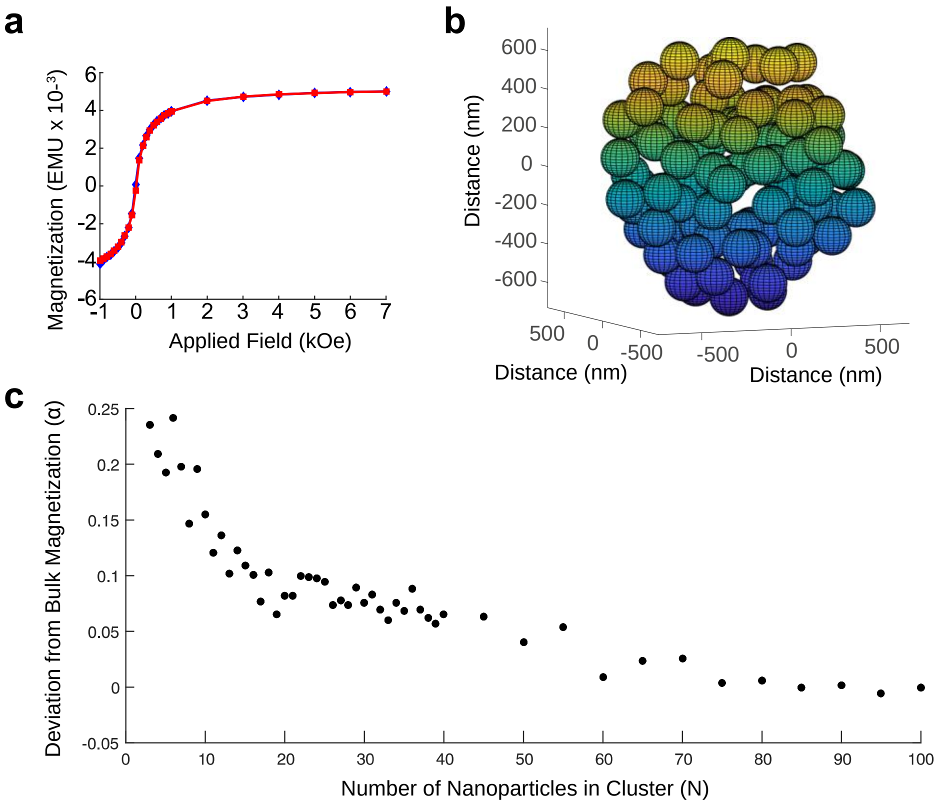

Supplementary Figure 3 - SQUID magnetometry and saturation of IONs (a) Superconducting quantum interference device (SQUID) magnetometry of a 100 µg stock of our IONs at 300K. **(b)** A representative pseudo-spherical cluster (N=100 nanoparticles) used in our Monte Carlo magnetization simulations. **(c)** Approximate error α of our bulk approximation for clusters containing varying numbers of nanoparticles. Each point represents the mean value from 60 random particle arrangements.

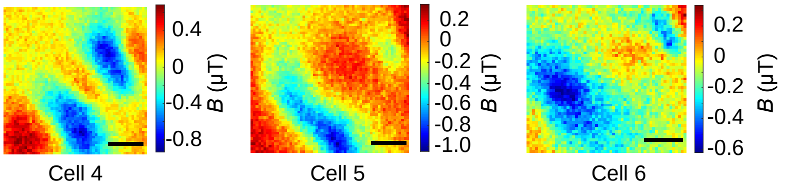

Supplementary Figure 4 - Additional cells for Monte Carlo library. Vector magnetometry results from three additional cells. These cells were measured as described in Figure 1 with the exception that the imaging time was cut to 2 hours. Scale bars are 2.5 μm.

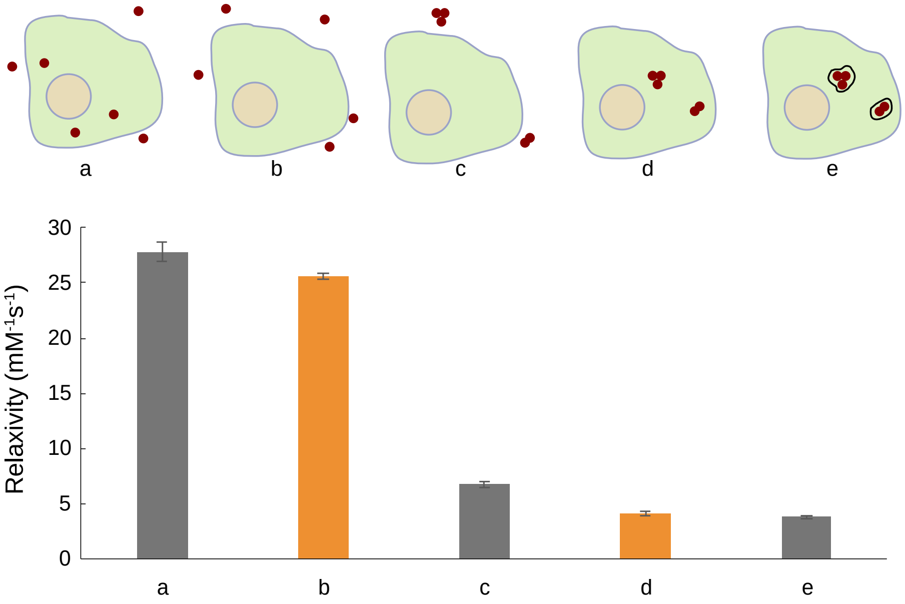

Supplementary Figure 5 - Supplementary *in silico* models of T_2 relaxation. In order to further assess the predicted effect of spatial frequency and cellular confinement on nanoparticle relaxivity, we simulated several additional particle distribution scenarios using the same Monte Carlo algorithm described in the main text. The scenarios are illustrated on top, with corresponding T_2 relaxivities below. Orange bars correspond to data in the main text. **(a)** Diffuse (unclustered) nanoparticles were randomly placed throughout the lattice. As this geometry minimized clustering, it maximized relaxivity for our IONs. **(b)** Unclustered particles randomly placed in the extracellular space in the lattice. This is the same as the diffuse condition that was experimentally verified in Fig. 2 of the main text. As the particles are still unclustered, the partial refocusing effect is small, maintaining the high nanoparticle relaxivity **(c)** Clusters from NV-established cell library randomly dispersed throughout the extracellular space of the lattice. The clustering of the particles significantly reduces their relaxivity relative to the unclustered condition, but the large distances between the clusters significantly increase relaxivity compared to clusters spatially confined in "host" cells, as shown in (d). **(d)** Cells from the NV library were randomly placed in the lattice, and clustered nanoparticles were confined inside of their host cells. This is the same condition as the "clustered" case that was experimentally verified in Fig. 2 of the main text. **(e)** In order to determine the effect of confinement in an intracellular compartment, we added an impermeable 5 nm diffusion barrier around the clusters and randomly placed them inside their host cells as in condition (d). There was a statistically insignificant decrease in the nanoparticle relaxivity, supporting the hypothesis that the majority of the relaxivity of these particles comes from is outer-sphere effects on aqueous protons. Error bars represent S.E.M.

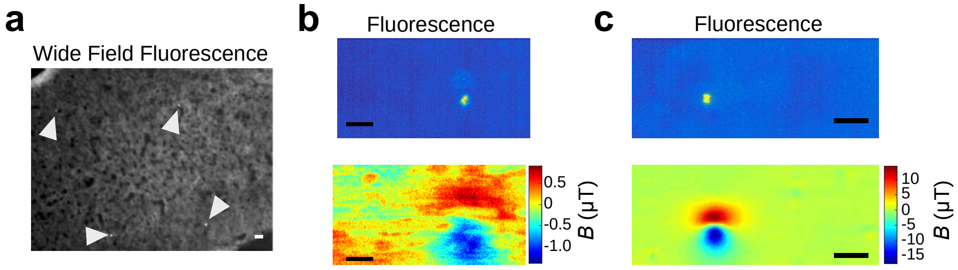

Supplementary Figure 6 - Additional tissue sections. (**a**) Fluorescent image of a wide field of view of a representative liver tissue section from an iron-injected mouse. Punctate fluorescent spots from the fluorescently labeled 900 nm ION are sparsely visible in the fluorescent image. (**b-c**) Field profile of two additional clusters measured using our NV microscope, measured as in Figure 2. Scale bars are 20 µm.

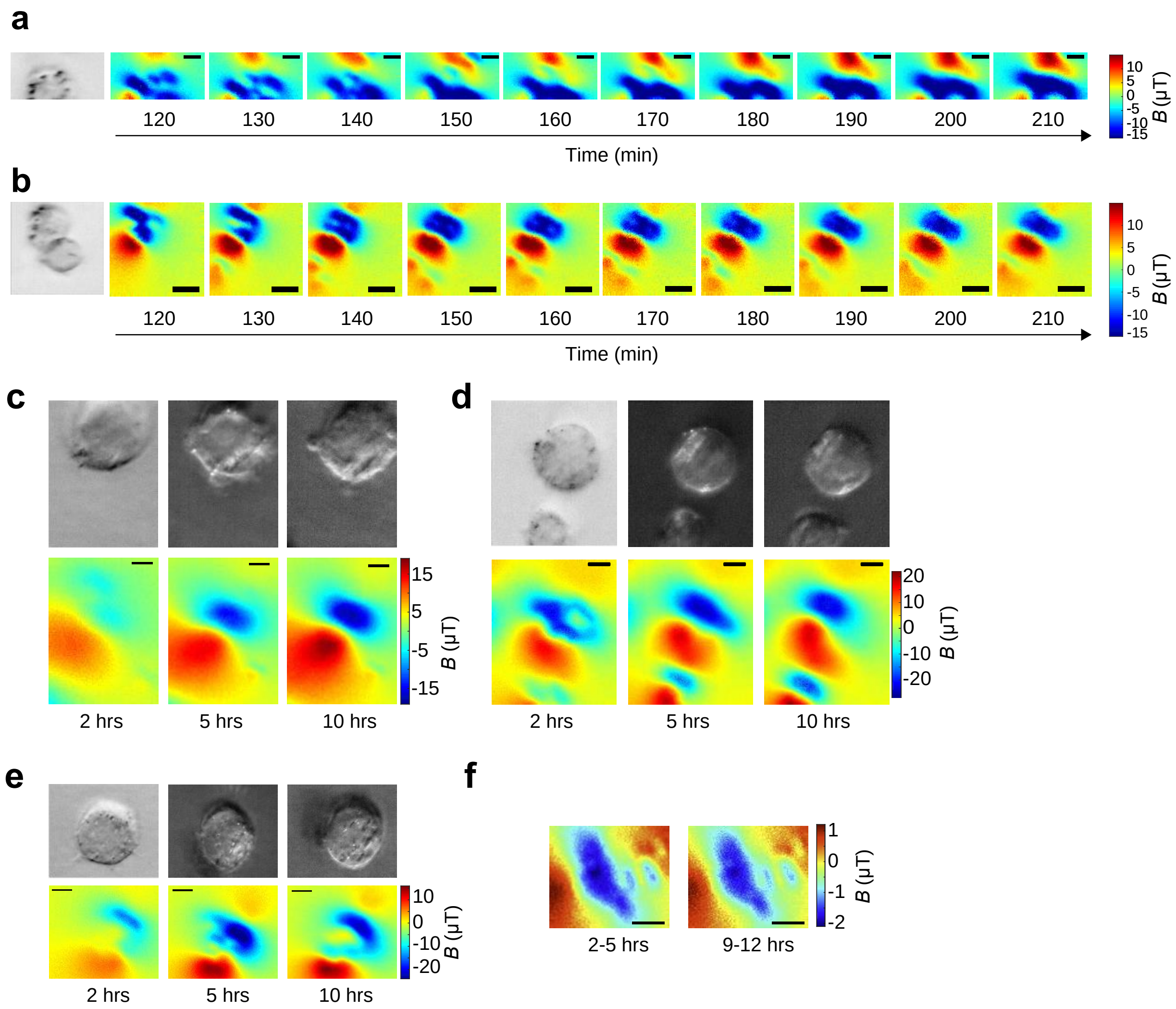

Supplementary Figure 7 - Live cell imaging with extended time course. (a–b) Two additional live cell replicates matching Figure 3b. Cells were confirmed alive with trypan blue after NV imaging. (**c–e**)

Bright field and magnetic images of ION endocytosis in RAW cells acquired 2, 5, and 10 hours after initial nanoparticle exposure to 279 ng ml^{-1} 200 nm IONs. Trypan blue assay revealed an ~70% viability for these imaging studies. All displayed cells were still alive after imaging. Bright field illumination was provided by a hand-positioned light emitting diode (LED) source that was repositioned between images. (**f**) Magnetic field maps from a single fixed cell acquired 7 hours apart to show the absence of dynamic changes in the magnetic field as a negative control for dynamic rearrangements seen in live cell experiments. Scale bars are 5 μm.

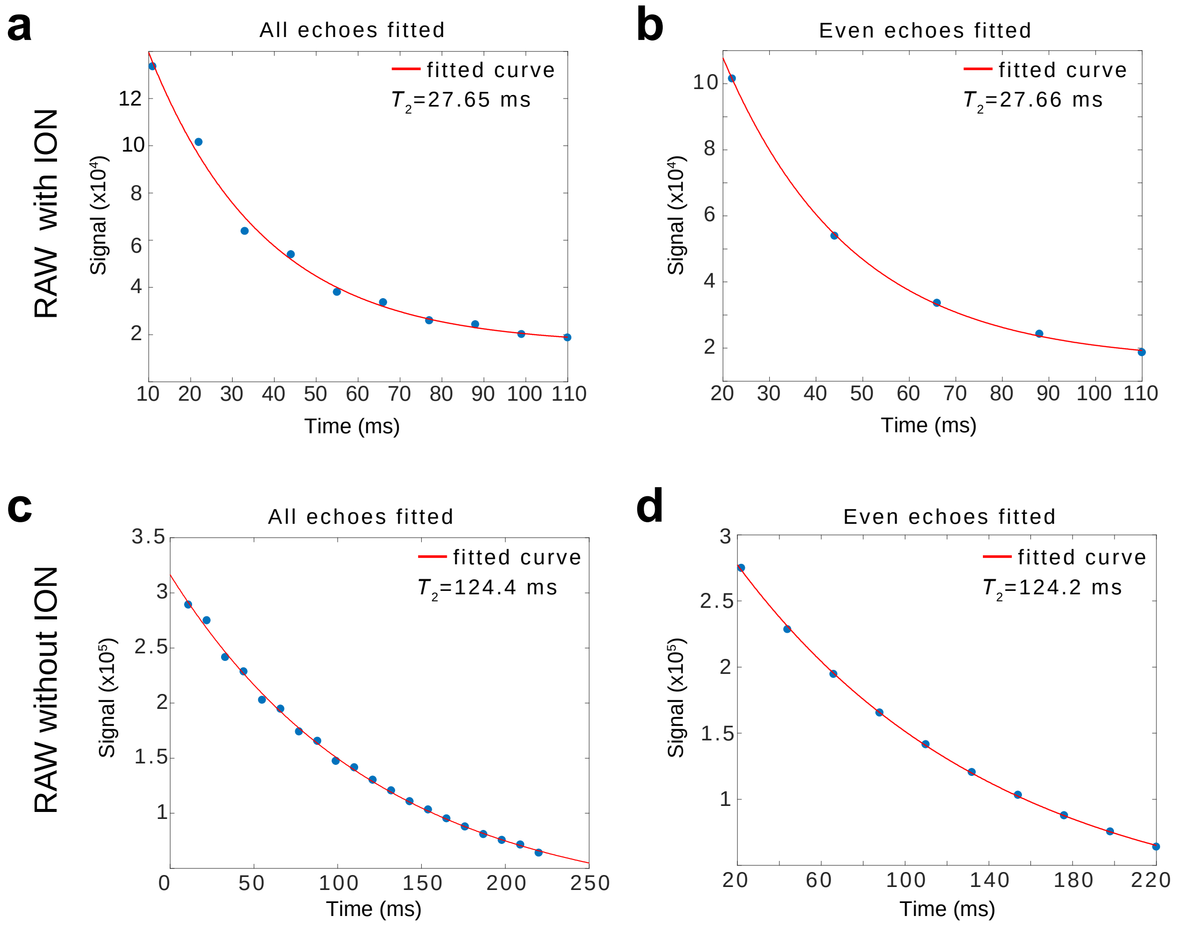

Supplementary Figure 8 - T_2 quantification. (a–b) The T_2 decay of a representative sample of ION-supplemented RAW cells with (a) the first 10 echoes used in fitting and (b) the same sample fitted with only the even echoes. **(c–d)** The T_2 decay of a representative sample of RAW cells with (c) the first 20 echoes used in the fitting and (d) the same sample fitted with only the even echoes. The T_2 value obtained from each fit is displayed on the plot.

Supplementary Note 1: SQUID Magnetometry and Saturation Field Scaling

Strong off-axis fields shift the eigenbasis of the Nitrogen Vacancy (NV) spin Hamiltonian from along the NV axis to along the applied field. In this condition, m_s is no longer an eigenstate of the spin Hamiltonian, leading to mixing of the m_s=0 and m_s=+/-1 states. This effect significantly reduces the sensitivity of NV vector magnetometry at bias fields above 10 mT [1]. Therefore, all our vector magnetometry experiments were conducted with a 10 mT bias field. To translate these measurements to the 7 T field strength of magnetic resonance imaging (MRI) in Monte Carlo simulations, we scaled the measured magnetic moments from the 10 mT bias field to 7 T using the results of superconducting quantum interference device (SQUID) magnetometry performed on a dried sample containing ~$3x10^9$ IONs (**Supplementary Fig. 3a**). This scaling works well for large pseudo-spherical clusters, but does not fully account for the difference in inter-particle effects between small clusters of nanoparticles and the dried SQUID sample in a non-saturated field. As has been previously demonstrated, bulk mass magnetization of continuum nanoparticle assemblies M_{Bulk} is reduced from the mass magnetization of a single nanoparticle or a small nanoparticle cluster (M) due to magnetic dipole coupling[2] such that:

$$M_{\text{Bulk}} = (1-\alpha)M \qquad (1)$$

To assess the potential impact of dipole-dipole interactions on the accuracy of our dipole scaling, we estimated it using a Monte Carlo model of magnetic coupling in nanoparticle clusters (**Supplementary Fig. 3b**). Since we used multi-core particles, we assumed that each nanoparticle has many domains and is in thermal equilibrium, allowing us to neglect the complex time-dependence of Neel relaxation for single domains. We spline-interpolated the SQUID magnetization curve in MATLAB, and solved the following many-body problem employing a similar method to one used previously to simulate magnetic dipole coupling [3]. Our governing equations are as follows:

$$\mathbf{m}_i = S\left[H_{\text{ef},i}\right] = S[\mathbf{H_0} + \textstyle\sum_{j\neq i}\mathbf{H}_j] \qquad (2)$$

$$\mathbf{H}_j = \frac{1}{4\pi}\left(\frac{3\mathbf{r}_{ij}(\mathbf{m}_j\cdot\mathbf{r}_{ij})}{r_{ij}^5} - \frac{\mathbf{m}_j}{r_{ij}^3}\right) \qquad (3)$$

Here m_i is the magnetic moment of the ith nanoparticle in the cluster, S$[H_{\text{ef},i}]$ is the splined approximation of the SQUID M vs H curve for the effective field at the location of the *i*th nanoparticle, $H_0 = 7958\ \text{A m}^{-1} = 100\ \text{Oe}$ is the initial bias field applied to all nanoparticles in the lattice, and $\boldsymbol{r}_{ij}$ separating the *i*th and the *j*th nanoparticle. The effect on the mass magnetization of clusters due to dipole coupling is calculated as follows: First, nanoparticles are randomly dispersed into a pseudo-spherical arrangement with packing fraction $\eta = 0.33$. This value is equal to the packing fraction from our Monte Carlo simulation and is within the range of measured values in literature [4,5]. Next, the magnetic moment of each nanoparticle is calculated based on of the bias field H_0. Next, the field at each nanoparticle is calculated as the superposition of the dipole fields from the other nanoparticles in the cluster. Next, in order to enforce a smooth process, the magnetic moment magnitude and orientation of each nanoparticle are adjusted to a weighted average of their value in the previous step and the value calculated from (**Supplementary Equation 2**). Next, the field calculation and magnetic moment adjustment steps are repeated until the effective applied field and the magnetic moment of each nanoparticle are aligned such that $\max_{i\epsilon[1,N]}\{\frac{\|\boldsymbol{m}_i\times\boldsymbol{H}_{\text{eff},i}\|}{\|\boldsymbol{m}_i\|}\} < 10^{-12}$ and the fractional change of each nanoparticle's magnetic moment is less than 10^{-15}. Finally, the dipole-coupling induced magnetization's deviation from the bulk measurement is quantified as:

$$\alpha(N) = \frac{(m_{\text{mc}}(N) - m_{\text{Bulk}}(N))}{m_{\text{mc}}(N)} \qquad (4)$$

Here $m_{\mathrm{mc}}(N)$ is the simulated magnetic moment of a cluster with N particles and $m_{\mathrm{Bulk}}(N)$ is the predicted magnetic moment for that cluster applying the bulk mass-magnetization. We assume that a 100-nanoparticle cluster behaves as bulk.

The results of this simulation are shown in **Supplementary Figure 3c**. The expected difference relative to the bulk measurement is largest for small clusters, where the dipole interaction is not in the continuum limit. However, in all cases it is below 25%, and for the mean cluster size of approximately 28 nanoparticles measured with our NV magnetometer it is 7.5%. The curve flattens above N=80, validating our treatment of N=100 as a bulk material. Overall, this represents a modest under-estimation of the mass magnetization, meaning that NV measurements would slightly over-estimate the mass of particles in a given cluster at 10 mT, which in turn would cause an over-estimation of the magnetic moment of a given cluster at saturation (7 T). This in turn could help to account for our simulation's approximately 3% over-estimation of the relaxivity of our clustered samples. Future work mapping magnetic fields of nanoparticles could use the presented simulations to better estimate the relaxivity from pseudo-spherical and anisotropic particle clusters.

Supplementary Note 2: Packing and Distribution Effects on T_2 Relaxivity

In **Figure 2** of the main text we evaluated the ability of NV measurement-based Monte Carlo modeling to predict the effect of nanoparticle clustering patterns in cells on T_2 relaxivity compared to unclustered particles distributed in the extracellular space. While these two cases enabled experimental validation of our method, we performed additional *in silico* trials of hypothetical particle geometries to better understand the parameters driving the measured difference in relaxation **(Supplementary Fig. 5)**.

One hypothetical condition (**Supplementary Fig. 5a**) addresses the significance of extracellular confinement for unclustered nanoparticles by randomly placing unclustered nanoparticles throughout the whole lattice, including intracellular space. Dispersing the particles throughout the entire lattice slightly increases their relaxivity compared to extracellular confinement, from 25.6 ± 0.3 $mM^{-1}s^{-1}$ to 27.8 ± 0.8 $mM^{-1}s^{-1}$. However, this effect is small compared to that caused by endocytosis and clustering (**Supplementary Fig. 5d**).

Two additional hypothetical conditions utilized clusters drawn from the NV-measured cell library described in the main text. One condition (**Supplementary Fig. 5c**) examined clustered nanoparticles placed in the extracellular, rather than intracellular, space. Clusters obtained from the NV measurement library were randomly distributed throughout the extracellular space of the cell lattice. This increased T_2 relaxivity from 4.1 ± 0.20 $mM^{-1}s^{-1}$ to 6.7 ± 0.3 $mM^{-1}s^{-1}$ compared to the cell-confined intracellular clusters analyzed in the main text. This 63% increase can be understood as arising from a more homogeneous distribution of particles in the lattice, compared to confinement within a subset of cells. This result supports the significance of using NV magnetometry to visualize the sub-tissue and sub-cellular distribution of magnetic fields.

The final condition analyzed the effect of confining intracellular clusters in a lipid compartment (**Supplementary Fig. 5e**). We simulated the effect of such a compartment by creating an impermeable 5 nm diffusion barrier surrounding the nanoparticle clusters. This decreased the relaxivity from 4.1 ± 0.20 $mM^{-1}s^{-1}$ to 3.8 ± 0.16 $mM^{-1}s^{-1}$, within statistical error, indicating that the majority of the contrast from these large nanoparticle clusters does not come from water molecules in close proximity to the cluster surface.

Supplementary Note 3: Uniqueness of Fit for Dipole Magnetization and Height off the Diamond

Here, we seek to demonstrate that for a given (z,**M**) value pair, there does not exist another (z',**M'**) value pair such that $B_x(x, y, z, \mathbf{M}) = B_x(x, y, z', \mathbf{M}')$ for all values of x and y. This can be proven by contradiction. The coordinate system is set such that the point dipole is at the origin and the measurement plane is below the point dipole and is parallel to the xy plane.

Assume there exists $(z, \mathbf{M})$ *and* $(z', \mathbf{M}'\ )$ *such that* $B_x(x, y, z, \mathbf{M}) = B_x(x, y, z', \mathbf{M}'\ )\ \forall\ (x, y)$

Let $(x, y) = (0, y_1)$ with $y_1 > 0$. From the equation for $B_x$provided in the main text,

$$\frac{\mathbf{M}\cdot\hat{x}}{r^3} = \frac{\mathbf{M}\prime\cdot\hat{x}}{r\prime^3} \tag{5}$$

Where $r_1 = \sqrt{{y_1}^2 + z^2}$ *and* $r_1' = \sqrt{{y_1}^2 + z'^2}$. Simplifying

$$\frac{M_x'}{M_x} = \frac{r_1'^3}{r_1^3} \tag{6}$$

Now take $(x, y) = (0, y_2)$ with $y_2 > 0$. By similar logic:

$$\frac{M_x'}{M_x} = \frac{r_2'^3}{r_2^3} \tag{7}$$

Where $r_2 = \sqrt{y_2^2 + z^2}$ *and* $r_2' = \sqrt{y_2^2 + z'^2}$. Substituting from equation S6

$$\frac{r_2'^3}{r_2^3} = \frac{r_1'^3}{r_1^3} \tag{8}$$

Plugging in the definitions and simplifying gives

$$\frac{y_1^2+z'^2}{y_1^2+z^2} = \frac{y_2^2+z'^2}{y_2^2+z^2} \tag{9}$$

Cross-Multiplying and Simplifying gives

$$z^2(y_1^2 - y_2^2) = z'^2(y_1^2 - y_2^2) \tag{10}$$

As the measurement plane is always below the magnetic source in our system, this implies either $z = z'$ *or* $y_1 = y_2$, both of which violate assumptions in the proof. Thus, sampling any two points with y>0 along x=0 on the measurement plane uniquely specifies both **M** and z. (It is trivial to demonstrate that this also holds for any two points with y<0. The degeneracy from $y_1 = -y_2$ makes sense given the symmetric shape of the dipolar field.) Due to signal-to-noise ratio (SNR) constraints and the need to localize the (x,y) position of the dipole source, we fit to many more than two points per dipole source.

Supplementary References

1. Tetienne, J.P. *et al.* Magnetic-field-dependent photodynamics of single NV defects in diamond: an application to qualitative all-optical magnetic imaging. *New Journal of Physics* **14**, 103033 (2012).
2. Sanchez, F.H.e.a. Dipolar interaction and demagnetizing effects in magnetic nanoparticle dispersions: introducing the Mean Field Interacting Superparamagnet Model (MFISP Model). *arXiv:1507.05192* (2015).
3. Varón, M. *et al.* Dipolar Magnetism in Ordered and Disordered Low-Dimensional Nanoparticle Assemblies. *Scientific Reports* **3**, 1234 (2013).
4. Wilhelm, C., Cebers, A., Bacri, J.C. & Gazeau, F. Deformation of intracellular endosomes under a magnetic field. *European Biophysics Journal* **32**, 655-660 (2003).
5. Aubertin, K. *et al.* Impact of photosensitizers activation on intracellular trafficking and viscosity. *PLoS One* **8**, e84850 (2013).
